# Supplementary material for: Single Cell Transcriptomic and Chromatin Profiles Suggest Layer Vb Is the Only Layer With Shared Excitatory Cell Types in the Medial and Lateral Entorhinal Cortex
Source: Front Neural Circuits. 2022 Jan 26;15:806154. doi: 10.3389/fncir.2021.806154 (PMC8826650; doi:10.3389/fncir.2021.806154)
Supplement: Supplementary file 14 [file Data_Sheet_1.ZIP › LEC_Sup_vs_MEC_Sup_Motifs/homerResults/motif14.similar.html]

motif14

## Information for motif14

C
G
T
A
C
G
T
A
C
G
T
A
A
G
T
C
C
G
T
A
C
G
T
A
C
T
G
A
G
A
T
C
A
G
T
C
A
C
T
G
  
Reverse Opposite:  

A
G
T
C
A
C
T
G
C
T
A
G
A
G
C
T
A
C
G
T
A
C
G
T
C
T
A
G
A
C
G
T
A
C
G
T
A
C
G
T
  

|  |  |
| --- | --- |
| p-value: | 1e-20 |
| log p-value: | -4.674e+01 |
| Information Content per bp: | 1.895 |
| Number of Target Sequences with motif | 3191.0 |
| Percentage of Target Sequences with motif | 23.93% |
| Number of Background Sequences with motif | 4104.3 |
| Percentage of Background Sequences with motif | 20.60% |
| Average Position of motif in Targets | 314.9 +/- 231.4bp |
| Average Position of motif in Background | 345.8 +/- 262.8bp |
| Strand Bias (log2 ratio + to - strand density) | -0.1 |
| Multiplicity (# of sites on avg that occur together) | 1.28 |
| Motif File: | file (matrix) reverse opposite |

### Similar de novo motifs found

|  |  |  |  |  |  |  |  |
| --- | --- | --- | --- | --- | --- | --- | --- |
| Rank | Match Score | Redundant Motif | P-value | log P-value | % of Targets | % of Background | Motif file |
| 1 | 0.611 | G C T A C G A T C T G A C G T A C T A G G T A C G T C A T C G A T G C A T C G A G T A C G T C A | 1e-16 | -38.912088 | 13.03% | 10.69% | motif file (matrix) |
| 2 | 0.630 | C T G A A C G T A C G T C G A T A C G T A C G T C G A T A C G T | 1e-9 | -21.659183 | 43.36% | 40.73% | motif file (matrix) |
| 3 | 0.642 | A G T C A C G T A C G T A C G T A C G T A C G T A C G T A C G T | 1e-7 | -17.868431 | 42.52% | 40.17% | motif file (matrix) |
| 4 | 0.725 | A C G T A C G T A C G T A C G T A C T G A C G T A C G T A C G T A C G T A C T G | 1e-3 | -8.715083 | 3.31% | 2.79% | motif file (matrix) |
| 5 | 0.661 | A C G T A C G T C G A T A C G T C G A T A C G T A C G T A C T G | 1e0 | -1.046805 | 9.17% | 9.08% | motif file (matrix) |
